# Polymorphic Control in Pharmaceutical Gel-Mediated Crystallization: Exploiting Solvent–Gelator Synergy in FmocFF Organogels

**DOI:** 10.3390/gels11070509

**Published:** 2025-07-01

**Authors:** Dong Chen, Koen Robeyns, Tom Leyssens, Basanta Saikia, Stijn Van Cleuvenbergen

**Affiliations:** 1 Department of Chemistry, Molecular Imaging and Photonics, KU Leuven, Campus KULAK Kortrijk, 8500 Kortrijk, Belgium; dong.chen@kuleuven.be (D.C.); bsaikia1@gmail.com (B.S.); 2 Institute of Condensed Matter and Nanosciences, Université Catholique de Louvain, 1348 Louvain-La-Neuve, Belgium; koen.robeyns@uclouvain.be (K.R.); tom.leyssens@uclouvain.be (T.L.)

**Keywords:** pharmaceutical polymorphs, FmocFF organogels, gel-mediated crystallization, crystal engineering, nilutamide, solvate, polymorph control

## Abstract

FmocFF is a highly versatile gelator whose π–π-stacking fluorenyl group and hydrogen-bonded peptide backbone permit gelation in a wide spectrum of solvents, providing a rich scaffold for crystal engineering. This study explores the synergistic effects of FmocFF organogels and solvent selection on controlling the polymorphic outcomes of nilutamide, a nonsteroidal antiandrogen drug with complex polymorphism. By systematically varying process parameters such as solvent type and concentration, we demonstrate remarkable control over crystal nucleation and growth pathways. Most significantly, we report the first ambient-temperature isolation of pure nilutamide Form II through acetonitrile-based FmocFF organogel, highlighting the unique interplay between solvent properties and gel fiber networks. Thermal analysis reveals that the organogel not only selectively templates Form II but also affects its thermal pathway. We also present compelling evidence for a new polymorph exhibiting second-harmonic generation (SHG) activity. This would represent the first non-centrosymmetric nilutamide form discovered, suggesting the gel matrix induces symmetry breaking during crystallization. We also characterize a previously unreported nilutamide–chloroform solvate through multiple analytical techniques including PXRD, DSC, FTIR, SXRD, and SHG microscopy. Our findings demonstrate that solvent-specific molecular recognition within gel matrices enables access to entirely new regions of polymorphic space, establishing gel-mediated crystallization as a broadly applicable platform technology for pharmaceutical solid form discovery under mild conditions.

## 1. Introduction

The choice of solid-state form for an active pharmaceutical ingredient (API) has a profound impact on its functionality and manufacturability. APIs can crystallize in a variety of forms—including polymorphs, solvates, hydrates, salts, and cocrystals—each offering distinct physicochemical profiles that affect properties such as solubility, dissolution, stability, and bioavailability [1,2]. Polymorphism, which refers to the ability of a compound to exist in more than one crystalline form, poses a critical challenge in drug development. The selection of an optimal solid form is therefore essential for ensuring therapeutic effectiveness and patient safety [3,4].

Supramolecular gel-phase crystallization has emerged as a promising strategy to manipulate nucleation and crystal growth behaviors [5,6,7,8]. Unlike conventional solvent systems, gels provide unique energy landscapes and microenvironments that are conducive to controlling the solid-state properties of APIs. These materials are formed via the self-assembly of low-molecular-weight gelators (LMWGs) through noncovalent interactions, constructing a fibrous network that entraps solvents [6,9]. Such confinement alters crystallization dynamics, promoting heterogeneous nucleation and influencing polymorph selection through epitaxial interactions [10,11]. Moreover, the gel matrix suppresses convective currents and bulk transport, yielding a diffusion-dominated environment favorable for growing high-quality single crystals [12].

Solvent selection remains a pivotal parameter influencing crystallization outcomes, as it directly affects API solubility, supersaturation profiles, nucleation kinetics, and ultimately polymorphic distribution [13,14,15,16,17]. The critical role of solvent in directing supramolecular self-assembly pathways has been extensively demonstrated, where solvent properties fundamentally alter assembly kinetics and resulting network architectures [18]. In gel-based systems, the ability to form gels in diverse organic and aqueous solvents expands the available crystallization platform. While hydrogels have been extensively studied, organogels (formed in non-aqueous solvents) are particularly well-suited for APIs with limited water solubility. The gelator–solvent combination dictates the resultant fiber morphology and network structure [19]. Gel-mediated crystallization benefits from cooperative interactions: solvent molecules participate in gelator assembly while simultaneously modulating API interactions. This dual role fosters unique supersaturation profiles and nucleation behaviors that are unattainable with solvent-only systems. The interplay between solvent and gel fibers enables crystallization pathways and polymorphs inaccessible by conventional means [11].

**Figure 1:**
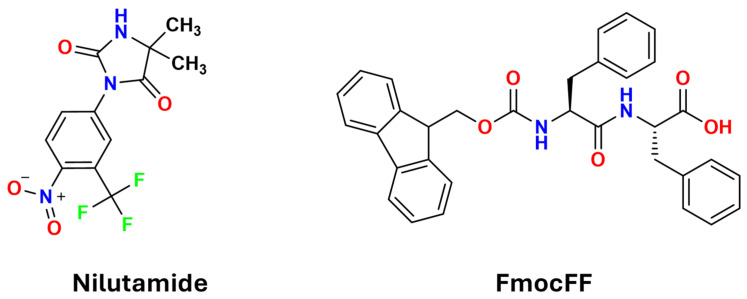
Molecular structures of nilutamide and FmocFF.

Building on these concepts, this study focuses on using FmocFF (9-fluorenylmethoxycarbonyl-phenylalanine-phenylalanine) organogels to control crystallization of nilutamide, a nonsteroidal antiandrogen used to treat metastatic prostate cancer (Figure 1) [20,21]. Nilutamide exhibits complex polymorphism, with five identified forms (Forms I–V) [22,23]. Although Form I is commercially available and most readily crystallizes under ambient conditions, obtaining pure metastable forms under mild conditions remains challenging. From a structural perspective, nilutamide and FmocFF possess complementary molecular recognition motifs: nilutamide features a hydantoin ring with one N–H donor and two C=O acceptors, whereas FmocFF contains amide, carboxyl, and carbamate groups acting as both donors and acceptors. Additionally, nilutamide contains an electron-deficient nitroaryl ring, while FmocFF includes large aromatic surfaces suitable for π–π stacking interactions through its fluorenyl and phenyl rings. Though primarily studied as a hydrogelator, FmocFF has recently attracted attention as a versatile organogelator, forming gels across diverse organic solvents and generating fibrous networks capable of interacting with API molecules [19].

In this work, we systematically explore the utility of FmocFF-based organogels to control nilutamide crystallization. By combining FmocFF with various organic solvents, diverse crystallization conditions were achieved, demonstrating synergistic effects between the gel matrix and solvent in regulating polymorphic outcomes. This strategy enabled the first ambient-temperature isolation of pure nilutamide Form II, the concomitant crystallization of multiple known polymorphs, and the potential discovery of a novel polymorph. Furthermore, we report the first single-crystal structure of a nilutamide–chloroform solvate. Solid-state characterizations including optical microscopy, powder X-ray diffraction (PXRD), differential scanning calorimetry (DSC), Fourier-transform infrared spectroscopy (FTIR), single-crystal X-ray diffraction, and second-harmonic generation (SHG) microscopy were conducted to comprehensively analyze the obtained polymorphs and solvates. These findings highlight the efficacy of combining gel-mediated crystallization and systematic solvent screening in achieving precise control over polymorphic outcomes, presenting a versatile approach for accessing elusive and novel solid forms of pharmaceutical compounds under ambient conditions.

## 2. Results and Discussion

### 2.1. Gel-Phase Crystallization of Nilutamide

**Figure 2:**
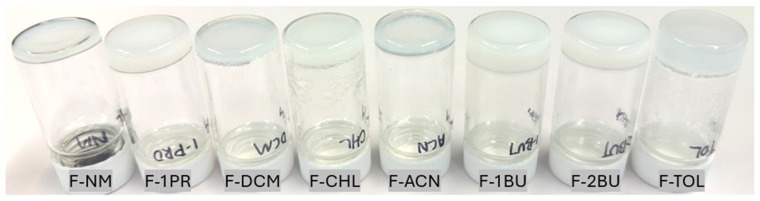
Images illustrating the outcomes of FmocFF organogel-mediated crystallization of nilutamide (C_Nil_@35 °C) conducted in eight different organic solvents (NM, 1PR, ACN, DCM, CHL, TOL, 1BU, 2BU) within 20 mL vials.

The ability of FmocFF to form organogels was assessed across a range of common organic solvents in previous work by some of us [19]. Stable and self-supporting gels were successfully formed in eight distinct solvents with different polarity: nitromethane (NM), 1-propanol (1PR), acetonitrile (ACN), dichloromethane (DCM), chloroform (CHL), toluene (TOL), 1-butanol (1BU), and 2-butanol (2BU). In order to induce gel-phase crystallization, supersaturated solutions of nilutamide were prepared at elevated temperatures of 25 °C and 35 °C, after which FmocFF was added, and the solution was cooled to room temperature. This facilitated crystallization in the gel matrix at room temperature within 2 to 5 days. The gelator concentrations applied here (Table 1) were slightly increased relative to the reported minimum gelator concentrations (MGCs) to ensure stable, self-supporting gels suitable for crystallization experiments. These concentrations ranged from 10 mg/mL in NM, DCM, and TOL, to 15 mg/mL in 1PR, ACN, and CHL, and up to 20 mg/mL in 1BU and 2BU, reflecting the influence of solvent properties on the self-assembly process of FmocFF in the presence of nilutamide. Importantly, control experiments demonstrated that pure FmocFF organogels, in the absence of nilutamide, produce no crystalline material. Figure 2 provides an overview of the nilutamide crystallization experiments conducted within these eight different FmocFF organogel systems (F-NM, F-1PR, F-ACN, F-DCM, F-CHL, F-TOL, F-1BU, F-2BU). Visual inspection revealed the formation of crystalline material in all gel systems, with noticeable variations in crystal size and morphology. These details are discussed in the following section.

### 2.2. Crystal Morphology and Habit Modification

**Figure 3:**
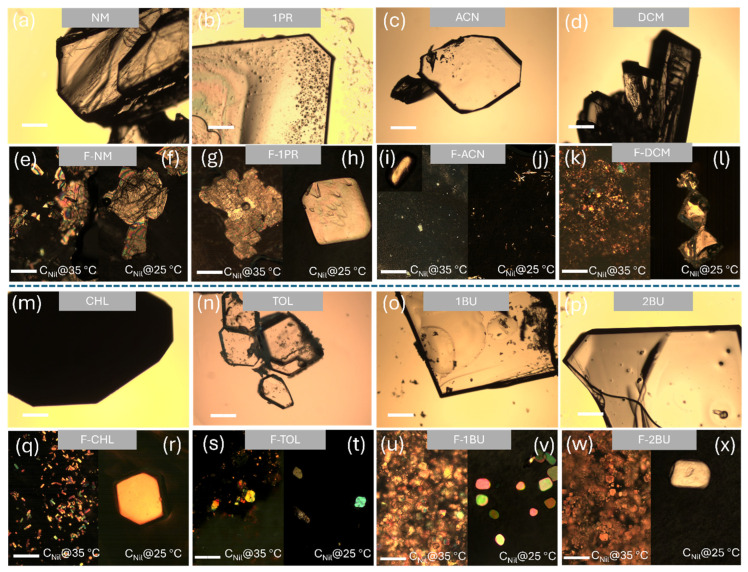
Comparative microscopy images. (Top row) Optical microscope images of nilutamide crystals obtained from evaporation of neat organic solvents (NM (**a**), 1PR (**b**), ACN (**c**), DCM (**d**), CHL (**m**), TOL (**n**), 1BU (**o**), 2BU (**p**)). (Bottom row) Polarized optical microscope images of nilutamide crystals formed within the corresponding FmocFF organogels (F-NM (**e**) and (**f**), F-1PR (**g**) and (**h**), F-ACN (**i**) and (**j**), F-DCM (**k**,**l**), F-CHL (**q**,**r**), F-TOL (**s**,**t**), F-1BU (**u**,**v**), F-2BU (**w**,**x**)); nilutamide under concentrations of C_Nil_@35 °C and C_Nil_@25 °C, respectively. Scale bar 200 μm.

Comparative morphological analysis using polarized optical microscopy revealed dramatic differences between solution- and gel-mediated crystallization (Figure 3). Crystallization from neat solvents generally yielded large block-like or flake-like crystals (Figure 3a–d,m–p), characteristic of typical morphologies of nilutamide Form I. Polar aprotic NM produced irregular thick blocks, while ACN and TOL produced prismatic blocks, DCM gave aggregated blocks, CHL grew wedge-shaped plates, and the alcohols (1PR, 1BU, 2BU) formed large prismatic plates. In contrast, gel-mediated crystallization consistently produced significantly smaller crystals (micrometer scale) with substantially higher nucleation densities. This size reduction results from enhanced heterogeneous nucleation on gel fibers combined with diffusion-limited growth within the confined gel matrix. For the gel-mediated crystallization, we employed polarized optical microscopy (POM) as the primary imaging modality, because the dense FmocFF network scatters bright-field light and blurs conventional images, whereas crossed polarizers suppress the fibrous background and sharply reveal the birefringent nilutamide crystals.

Most significantly, the supersaturation level critically influenced crystallization outcomes in acetonitrile. For C_Nil_@35 °C, POM images revealed birefringent crystalline material within the gel networks (Figure 3e,g,i,k,q,s,u,w). Solvent–gel synergy, rather than the gelator alone, dictated the emergent habit: small blocks in F-NM and irregular plates in F-1PR, dense small block-crystallites in F-DCM, small elongated platelets in F-CHL, irregular platelets and block-like crystals in F-TOL, and dense platelets in the viscous 1BU/2BU gels. Specifically, supersaturation further modulated these patterns. At the higher loading (C_Nil_@35 °C), every gel exhibited far more nuclei and correspondingly finer crystallites. In contrast, lowering the supersaturation to 25 °C resulted in fewer, larger crystals. The only qualitative habit switch occurred in acetonitrile: F-ACN under C_Nil_@35 °C produced smaller-size block-like crystals (Figure 3i and the inserted zoomed-in image). When the concentration of nilutamide was reduced to C_Nil_@25 °C, distinct needle-like crystals were formed within the F-ACN gel, alongside smaller block-like crystals, as shown in the polarized microscope image in Figure 3j. These needle-like crystals are believed to be a new solid form based on their SHG activity (details below).

This suggests that kinetic factors, modulated by the interplay between concentration, solvent environment, and the FmocFF gel network, play a crucial role in directing nucleation and crystal growth pathways in this system. This finding points towards the possibility of accessing novel crystalline forms or habits by carefully tuning parameters within the gel environment. Another noteworthy observation occurred during crystallization from pure chloroform (CHL). Initially, transparent, well-defined crystals formed. However, upon removal from the mother liquor, these crystals underwent a transformation, becoming opaque and polycrystalline, as depicted in Figure S1. This behavior is characteristic of desolvation, suggesting the initial formation of a transient nilutamide–CHL solvate that subsequently converts to a non-solvated form upon solvent loss. The same solvate also arises in the F-CHL gel, where the radiating needle habit indicates strong nilutamide–chloroform interactions before desolvation. Taken together, these observations indicate that (i) gel mediation converts convection-controlled growth into diffusion-limited, template-assisted nucleation, (ii) systematic solvent screening within the gel framework appears as a powerful lever for tuning crystal habit and uncovering novel forms, and (iii) fine-tuning supersaturation can be decisive in stabilizing polymorphic forms through gel-mediated crystallization. These observations provide the basis for the detailed structural, thermal, and spectroscopic analyses presented in the following sections.

### 2.3. Polymorphic Identification and Novel Form Discovery

**Figure 4:**
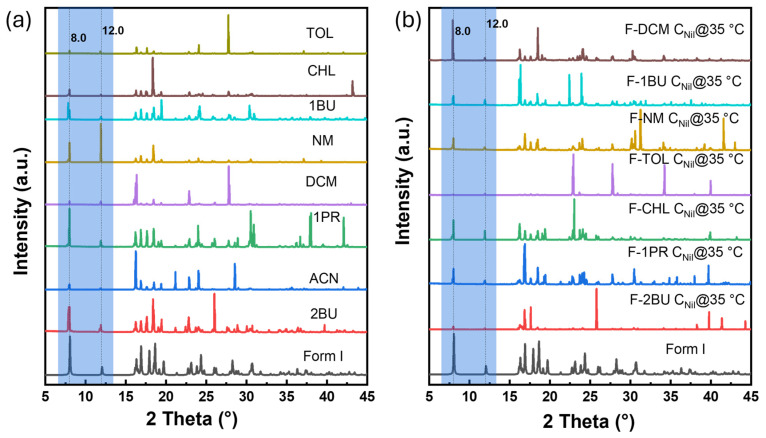
Powder X-ray diffraction (PXRD) patterns of nilutamide crystals obtained from evaporation of eight different neat organic solvents (**a**) and crystals obtained from crystallization within seven FmocFF organogels under C_Nil_@35 °C (**b**). All samples were vacuum-filtered and dried in fume hood at room temperature. The light blue shaded region indicates the key discriminating range for nilutamide.

To date, five polymorphs of nilutamide (Forms I–V) have been reported. Form I is thermodynamically stable at ambient conditions, whereas Forms II and III are usually obtained only concomitantly. Forms IV and V have thus far been reported structurally only from concomitant crystallization in mixed solvents. However, the authors subsequently could not reproduce these results, citing poor reproducibility of the crystallization protocol [23]. The five reported polymorphs (Form I to V) can be distinguished by their discriminating powder X-ray diffraction (PXRD) peaks between 5° and 15° 2θ (Figure S2). Figure 4a compares the PXRD patterns for the crystals obtained from solution crystallization and those from FmocFF-organogel crystallization. Solution crystallization from neat NM, 1PR, ACN, CHL, DCM, TOL, 1BU, and 2BU invariably yielded Form I, characterized by diffraction peaks at ca. 8.0° and 12.0° 2θ. In CHL, a transient solvate precipitated first, which after solvent loss converted to Form I before PXRD measurement.

**Figure 5:**
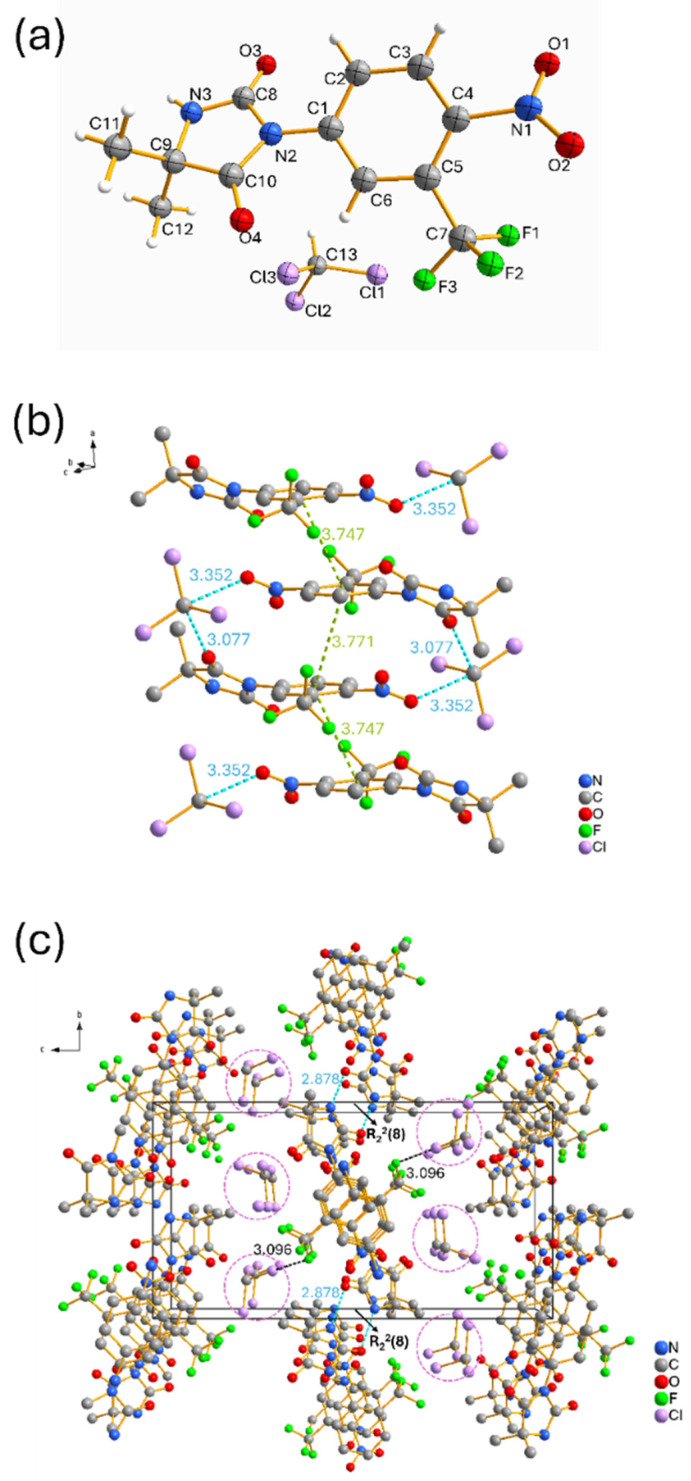
(**a**) The asymmetric unit of nilutamide·CHCl_3_ solvate, shown with the atom-labelling scheme; displacement ellipsoids are drawn at the 50% probability level. (**b**) Part of the crystal packing of nilutamide·CHCl_3_ solvate; nilutamide molecules stack each other through π–π interaction (green dash lines) and hydrogen bonds (blue dash lines). (**c**) View of the 3D framework structure of nilutamide·CHCl_3_ solvate, which is connected by hydrogen bonds (blue dash lines) and the weak halogen–halogen interaction (black dash lines). The solid black rectangle traces the edges of a single crystallographic unit cell. Purple dash circles point out the solvent channels. H atoms have been omitted for clarity.

Upon screening the solid form in FmocFF organogels (Figure 4b and Figure S3), PXRD patterns for nilutamide recovered from F-NM, F-1PR, F-DCM, F-TOL, F-1BU, and F-2BU also matched Form I. In F-CHL, we again observed the transient solvate. To confirm its identity, we isolated single crystals suitable for single-crystal X-ray diffraction (SXRD). Structure solution showed the space group of *P*2_1_/*n*, revealing a novel nilutamide–chloroform solvate with a 1:1 stoichiometry in the asymmetric unit. The ORTEP diagram for the nilutamide·CHCl_3_ solvate is shown in Figure 5a. The nilutamide molecule maintains its expected molecular conformation with the imidazoline ring adopting a near-planar geometry [22]. In the crystal packing, the nilutamide molecules stack through π–π interactions between the nitroaryl rings (Figure 5b) in an alternating way with interplanar distances of 3.747 Å and 3.771 Å, respectively. These π-stacking interactions provide directional assembly along one dimension. Concurrently, electron-deficient carbon atoms in chloroform form C-H···O hydrogen bonds with the oxygen atoms of nilutamide molecules, more likely with the oxygen atom from the carbonyl group (3.077 Å) than that from the nitro group (3.352 Å). These hydrogen bonds, though relatively weak compared to conventional ones, play a crucial role in the solvate formation. The combined effect of these interactions generates infinite one-dimensional ribbons that propagate along the a-axis. As shown in Figure 5c, these one-dimensional ribbons are further interconnected by amide dimers of R_2_^2^ (8) motifs through N–H···O hydrogen bonds (2.878 Å), as well as the weak halogen–halogen interaction (3.096 Å) between F and Cl atoms.

The three-dimensional framework reveals a notable structural feature: the presence of solvent channels (Figure 5c), which accommodate the chloroform molecules within the crystal lattice. While the channels provide the necessary void space for solvent inclusion, they paradoxically contribute to the solvate’s instability by creating continuous diffusion pathways rather than discrete binding pockets. The C-H···O hydrogen bonds between chloroform and nilutamide oxygen atoms create interaction motifs that insufficiently anchor the solvent molecules within the channels. These realities enable facile migration of chloroform molecules even under ambient conditions. Desolvation of this solvate leads to Form I as evidenced by the XRPD pattern shown in Figure 4.

CCDC 2454538 includes the supplementary crystallographic data and can be downloaded free of charge from the Cambridge Crystallographic Data Centre via www.ccdc.cam.ac.uk/structures (accessed on 27 May 2025).

**Figure 6:**
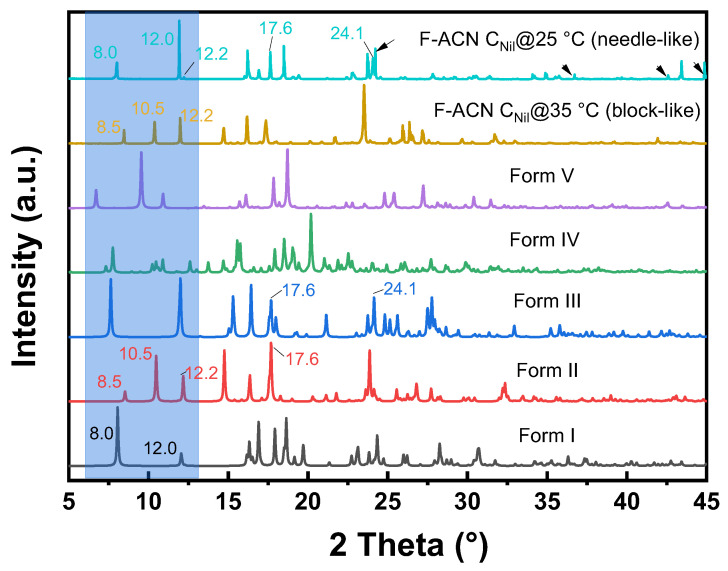
PXRD patterns of five different nilutamide polymorphs and the crystals obtained from F-ACN with different equilibrium concentrations, with arrows pointing out unique peaks in F-ACN C_Nil_@25 °C. The light blue shaded region indicates the key discriminating range for nilutamide.

**Figure 7:**
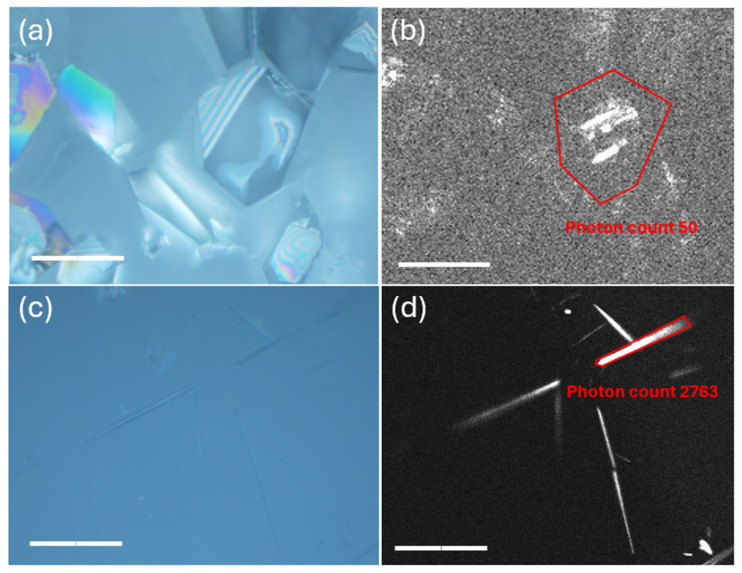
Optical microscope image of nilutamide Form I (**a**) and the corresponding SHG image (**b**), as well as an optical microscope image of nilutamide from F-ACN C_Nil_@25 °C (**c**) and the corresponding SHG image (**d**); red-circled areas represent the measured single crystals and the corresponding SHG signal by photon count. All with scale bar 50 μm.

Gel-mediated crystallization from ACN revealed significant differences from neat solvent crystallization (Figure 6). At high supersaturation (C_Nil_@35 °C), the gel yielded pure nilutamide Form II, with characteristic peaks at ca. 2θ of 8.5°, 10.5°, and 12.2°. FTIR further confirmed that crystals formed in F-ACN belong to Form II (Figure S4) [22]. This is, to our knowledge, the first direct isolation of pure nilutamide Form II at ambient temperature. Previous approaches to isolate Form II have relied on techniques such as lyophilization or melt crystallization [22]. Reducing the supersaturation (C_Nil_@25 °C) produced a mixture of both needle- and block-like crystals. PXRD shows reflections of Forms I (8.0°, 12.0°), II (12.2°, 17.6°), and III (17.6°, 24.1°) together with four additional peaks (24.2°, 36.7°, 42.6°, 44.9°) that cannot be indexed to any known form or to the gelator (PXRD of neat FmocFF, Figure S5). These extra reflections indicate the emergence of a yet unreported polymorph. Multiple attempts to obtain suitably sized single crystals from the needle-like crystals that uniquely form in the ACN organogel were unsuccessful. Additional characterization was therefore undertaken using SHG microscopy. SHG microscopy has emerged as a powerful tool for discriminating centrosymmetric from non-centrosymmetric materials, with applications spanning from crystallographic characterization to real-time monitoring of nucleation processes [24]. The technique enables comprehensive structural analysis of organic crystals through optical means [25] and has proven particularly effective for probing the relationship between molecular organization and bulk nonlinear optical responses in various systems [26]. The needles show a strong SHG response (Figure 7a,b) whereas block-like crystals are SHG-silent (Figure 7c,d). This finding provides compelling evidence for a novel nilutamide polymorph, as all previously reported forms (I–V) crystallize in centrosymmetric space groups and are therefore SHG-inactive. For comparison, we also performed SHG imaging on Form I crystals, which—as expected for a centrosymmetric phase—were largely SHG-inactive. In some cases, weak SHG signals were detected, but these were confined to localized regions within the crystals and were consistently more than 55-fold weaker than those observed for the needle-like crystals. Such residual SHG activity likely arises from structural imperfections or grain boundaries where bulk centrosymmetry is locally disrupted. Nonetheless, the overall absence of bulk SHG response confirms the centrosymmetric nature of Form I and highlights the marked contrast with the non-centrosymmetric needle-like crystals. This finding therefore suggests that the needle-like material represents a novel nilutamide polymorph formed under conditions of reduced supersaturation.

### 2.4. Thermal Analysis and Gelator–API Interactions

**Figure 8:**
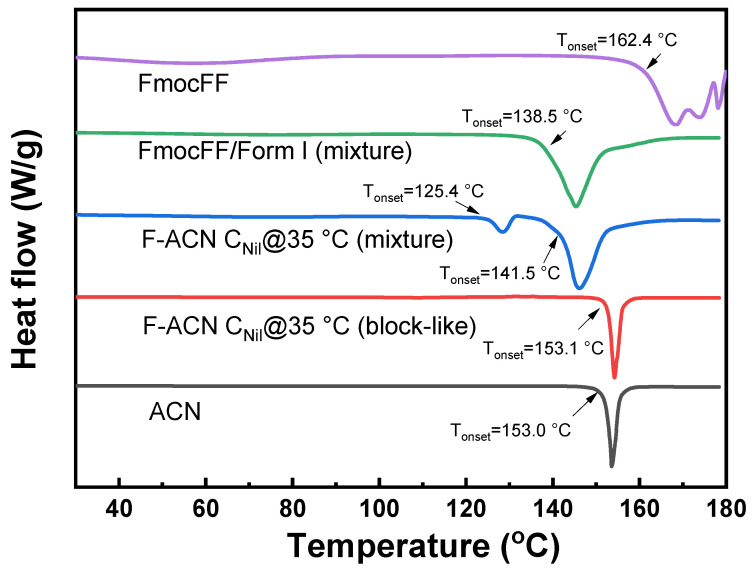
Differential scanning calorimetry (DSC) thermogram of compounds, including ACN crystallized nilutamide (ACN), gel-phase crystallized F-ACN C_Nil_@35 °C with vacuum filtration and rinsing (F-ACN C_Nil_@35 °C (block-like)), gel-phase crystallized F-ACN C_Nil_@35 °C only with vacuum filtration (F-ACN C_Nil_@35 °C (1/5 mixture on preparation)), physically triturated FmocFF and Form I with mass ratio 1:5 (FmocFF/Form I (1/5 mixture)), and FmocFF raw material (FmocFF).

To compare the thermal behavior of the alternative solid forms obtained via gel-mediated crystallization with that of the stable nilutamide Form I, we performed DSC at a scan rate of 10 °C min^−1^ on selected samples, including material crystallized from pure ACN and from FmocFF-based ACN organogels (Figure 8). Form I recrystallized from neat ACN (black curve) shows a melting point onset at 153.0 °C, in close agreement with the reported 153.3 °C [22]. Earlier literature reported two thermal events for pure Form II: a minor exothermic transition at 125 °C, attributed to the Form II → Form I transformation, followed by melting at 153.3 °C.

In our study, the sample recovered directly from the ACN organogel that was identified as Form II through PXRD and FTIR was vacuum filtered without rinsing (blue curve). The sample therefore contained residual FmocFF fibers (1/5 mass ratio of FmocFF/nilutamide on preparation). It displays two thermal events: an endotherm that begins at 125.4 °C and a typically broad shoulder at 141.5 °C. While pure FmocFF (purple curve) shows a structured broad endotherm with onset at 162.4 °C, the two thermal events can be explained as eutectic at 125.4 °C followed by a liquidus melting. Meanwhile, we measured a control trace in which FmocFF was physically triturated with Form I in a similar 1/5 ratio (green curve), which however only exhibits a single liquidus appearing at 138.5 °C. The missing eutectic could result from the improper heating rate under which the small eutectic signal has been covered. The traces make clear that the organogel not only selects Form II at room temperature but also reshapes its entire thermal pathway. From the above, we can infer that the interactions between nilutamide Form II and FmocFF developed during the gel-phase crystallization. In another test, Form II collected from the organogel system with rinsing (red curve) displays a melting point identical to that of Form I, yet no distinct exothermic transition corresponding to the Form II → Form I transformation is observed. This absence may be attributed to minor impurities present within the sample, which could obscure the phase transition.

Considering only F-ACN results in the formation of alternative solid forms, the role of acetonitrile as the proper solvent must not be neglected. Interestingly, it has been reported that a concomitant crystallization of Form I/Form II can be achieved through an acetonitrile/water mixed solution [22], in which acetonitrile’s unique combination of moderate polarity and aprotic character creates an opportunity to stabilize the nucleation of Form II. Therefore, the F-ACN uniquely provides a solvent–gel balance that further templates and stabilizes the Form II structure, whereas other gels do not, highlighting the powerful interplay between solvent environment and gel-mediated polymorph control.

## 3. Conclusions

This study demonstrates that FmocFF organogels provide a versatile platform for controlling nilutamide crystallization, enabling selective access to four distinct solid forms through systematic variation of solvent identity and supersaturation level: stable Form I, the first room-temperature isolation of pure Form II, a novel nilutamide·CHCl_3_ solvate, and a suspected SHG-active polymorph. The formation of an SHG-active (non-centrosymmetric) nilutamide polymorph is noteworthy given that all five previously reported forms crystallize in centrosymmetric space groups. This finding shows that gel matrices can fundamentally alter crystallization pathways to access entirely new regions of polymorphic space that remain inaccessible through conventional solution-based methods. This principle of gel-induced symmetry breaking could prove transformative for discovering non-centrosymmetric pharmaceutical polymorphs with potentially enhanced properties, and may find applications extending beyond pharmaceuticals into photonic and electronic systems [27]. The solvent-dependent polymorphic outcomes underscore the critical synergy between the gel fibers and solvent environment in reshaping crystallization kinetics by altering interfacial binding, desolvation, and confinement effects. Our optical, structural, and thermal data are consistent with the view that solvent retained in the fibrous mesh can locally affect supersaturation gradients. These gradients, together with possible templating by fiber surfaces, may bias the system toward early kinetic products that subsequently evolve toward near-equilibrium polymorphs. Although these findings are based on a limited dataset, they indicate that systematic process variable optimization can unlock new polymorphic landscapes even with well-established gelators, offering a tractable route for accessing elusive pharmaceutical solid forms without requiring complex gelator design. Apparently, we cannot claim parity with purpose-designed functional gelators, but the results point to a tractable, chemistry-neutral route that warrants deeper investigation. Future studies should integrate experimental screening with in situ calorimetry, variable-temperature diffraction, and molecular simulation to better quantify the intermolecular forces governing gel-induced polymorph selection and to facilitate the rational discovery and stabilization of new pharmaceutical solid forms.

## 4. Materials and Methods

### 4.1. Materials

Fmoc-Phe-Phe-OH (FmocFF, 95%) was purchased from abcr GmbH (Karlsruhe, Germany). Nilutamide (98%) was purchased from Sigma-Aldrich (Darmstadt, Germany). Dichloromethane (DCM, 99%) and chloroform (CHL, 99%) were purchased from Fisher Scientific Company (Heysham, UK). 1-Butanol (1BU, 99%), 1-propanol (1PR, 99%), 2-butanol (2BU, 99%), acetonitrile (ACN, 99%), nitromethane (NM, 99%), and toluene (TOL, 99%) were purchased from Sigma–Aldrich (Buchs, Switzerland). All the reagents were used without further purification.

### 4.2. Gel Preparation

#### 4.2.1. Gel-Mediated Crystallization

For gel-phase crystallization of nilutamide in organogels, stable supersaturated nilutamide solutions were prepared in each of the eight selected solvents (NM, 1PR, ACN, DCM, CHL, TOL, 1BU, 2BU) by dissolving an excess amount of nilutamide under heating to either 35 °C or 25 °C, followed by filtration with preheated syringes at the same temperature (C_Nil_@35 °C and C_Nil_@25 °C, respectively). An appropriate amount of FmocFF was dissolved in 1 mL of the selected nilutamide solution in a 20 mL vial, followed by sonication at 40 °C until the solid fully dissolved. The closed vials were left undisturbed at room temperature (22 °C) and checked for gelation after cooling down, simply by turning the recipient upside-down. Three to six replicate vials were prepared for each condition, and nilutamide crystals were obtained within 2–5 days.

To harvest crystals from the organogels, the entire gel phase was vacuum-filtered and rinsed with appropriate amounts of toluene.

#### 4.2.2. Solution Crystallization

For comparison, control crystallization experiments were performed from neat organic solvents. Supersaturated solutions of nilutamide were prepared in each of the eight solvents (NM, 1PR, ACN, DCM, CHL, TOL, 1BU, 2BU) by dissolving an excess amount of nilutamide with heating at 35 °C, followed by filtration. The clear supersaturated solutions were allowed to evaporate slowly at room temperature (22 °C) in loosely covered vials, leading to crystal formation. The resulting crystals were collected by filtration.

#### 4.2.3. Empirical Solubility

The solubility of nilutamide in various solvents was determined using a stepwise dissolution method. Vials containing a fixed amount of nilutamide (5–25 mg) were gradually supplemented with 25 μL increments of the corresponding solvent until complete dissolution was observed. During this process, the vials were sealed, stirred continuously, and incubated in a thermal bath maintained at either 25 °C or 35 °C. The obtained empirical solubility is listed in Table S1.

### 4.3. Characterizations

#### 4.3.1. Polarizing Optical Microscopy (POM)

Polarizing optical microscopy (POM) was employed to examine the morphological features of the samples. Observations were carried out under both polarized and non-polarized light conditions using an Olympus BX51 microscope (Tokyo, Japan).

#### 4.3.2. X-Ray Powder Diffraction (XRPD)

X-ray Powder Diffraction (XRPD) patterns were obtained on a Malvern PANalytical Empyrean diffractometer (Malvern, UK) equipped with a PIXcel3D solid-state detector using a Cu anode (Cu Kα1: 1.5406 Å; Cu Kα2: 1.5444 Å). The powders and gels dried at different conditions were loaded onto a 96-well sample holder and X-ray diffractograms were recorded at room temperature within a 5–45° 2θ range using a step size of 0.013°.

#### 4.3.3. Single-Crystal X-Ray Diffraction (SXRD)

Diffraction data were collected on a MAR345 image plate detector (MAR Research, Norderstedt, Germany) and Mo Kα radiation (λ = 0.71073 Å) produced by an Incoatec IµS microfocus source. Data integration and reduction were performed by CrystAlis^PRO^ [28]. The structures were solved using the dual-space algorithm in SHELXT [29] and further refined against *F*^2^ using SHELXL [29]. All non-hydrogen atoms were refined anisotropically and C-H hydrogen atoms were placed at calculated positions and refined in riding mode, with temperature factors set at 1.2 U_eq_ of the parent atoms. The hydrogen-bonded N-H hydrogen was located in the difference map and refined in riding mode with refined N-H distance.

#### 4.3.4. Fourier-Transform Infrared Spectroscopy (FTIR)

FTIR spectra were recorded using a Bruker Alpha II spectrometer (Billerica, MA, USA). Each sample was analyzed in the form of a pressed pellet, prepared by compressing 1 mg of sample with 200 mg of KBr under a pressure of 9 t for 30 s using a manual hydraulic press. Spectra were collected over the range of 4000–400 cm^−1^ at a resolution of 4 cm^−1^, averaging 16 scans per sample. Background spectra were recorded before each measurement and automatically subtracted to ensure baseline correction.

#### 4.3.5. Differential Scanning Calorimetry (DSC)

Differential scanning calorimetry (DSC) was carried out using TA-DSCQ2000 (New Castle, DE, USA) at a single heating rate of 10 °C/min from 25 °C to 180 °C, under purging gas N_2_ with a flow rate of 50 mL/min. All samples (around 5 mg each) were non-hermetically sealed in aluminum crucibles (Tzero Lids, New Castle, DE, USA).

#### 4.3.6. Second-Harmonic Generation Microscopy (SHG Microscopy)

For second-harmonic generation (SHG) imaging, a self-built optical platform was applied as described in the work by de Coene et al. [30]. The sample was prepared by using a cavity slide (BRAND, Wertheim, Germany) and sealed with a cover glass; the edge of the cover glass was further sealed by silicon oil to prevent desolvation. Irradiation with a femtosecond pulsed infrared (IR) laser at 1030 nm (Pharos, Light Conversion, Vilnius, Lithuania) ensued. The intensity and polarization of the incident IR light was varied by a combination of a zero-order half-wave plate (Thorlabs, Newton, NJ, USA) for 1030 nm, mounted in a computer-controlled rotation stage (Thorlabs, PRM-Z8, rotation stage (Thorlabs, PRM-Z8), and a Glan–Taylor polarizer selecting for S-polarized light. A Thorlabs MM101 Multiphoton Microscope (Sterling, VA, USA) was used for scanning SHG imaging as well as for the brightfield imaging. A 20× objective (Olympus, 0.5 NA, Tokyo, Japan) focused the laser light and collected SHG in epi configuration. The incident laser power was measured and fixed at 23 mW after the objective, the Pixel Dwell Time at 2.5 μs and PMT gain 20. The generated signal was selected by a filter (Thorlabs, Bandpass 515 nm) for SHG imaging.

#### 4.3.7. Calculation Methods

Simulated PXRD patterns were obtained from the nilutamide single-crystal structures deposited in the Cambridge Structural Data (CSD) [31] using Mercury software (2021.3.0) [32], with refcodes LANLET [22], LANLET01 [22], LANLET02 [22], LANLET03 [23], and LANLET04 [23] for Forms I, II, III, IV, and V, respectively.

## Figures and Tables

**Table 1:** FmocFF concentrations in eight different organogels for organogel-mediated crystallization of nilutamide.

Organogel Label	Solvent	FmocFF Concentration (% *w*/*v*)
F-NM	Nitromethane	10
F-1PR	1-Propanol	15
F-ACN	Acetonitrile	15
F-DCM	Dichloromethane	10
F-CHL	Chloroform	15
F-TOL	Toluene	10
F-1BU	1-Butanol	20
F-2BU	2-Butanol	20

## Data Availability

The original contributions presented in this study are included in the article/Supplementary Materials. Further inquiries can be directed to the corresponding author(s).

## Supplementary Materials

The following supporting information can be downloaded at: https://www.mdpi.com/article/10.3390/gels11070509/s1, Table S1: Empirical solubility of nilutamide in different organic solvents at 25 °C and 35 °C (mg/mL); Figure S1: Optical images illustrating the crystal transformation observed for nilutamide crystallized from neat chloroform (CHL); Figure S2: PXRD patterns of five reported nilutamide polymorphs (Form I to V), the blue block highlight distinguishable fingerprint peaks between 2θ value 5° and 15°; Figure S3: Powder X-ray Diffraction (PXRD) patterns of nilutamide crystals obtained from crystallization within seven FmocFF organogels under C_Nil_@25 °C; Figure S4: FTIR of nilutaimide Form I and II; Figure S5: PXRD patterns of commercially available FmocFF and nilutamide from F-ACN C_Nil_@25 °C.

## References

[1] Chen J., Sarma B., Evans J.M.B., Myerson A.S. (2011). Pharmaceutical Crystallization. Cryst. Growth Des..

[2] Lee E.H. (2014). A Practical Guide to Pharmaceutical Polymorph Screening & Selection. Asian J. Pharm. Sci..

[3] Gao Z., Rohani S., Gong J., Wang J. (2017). Recent Developments in the Crystallization Process: Toward the Pharmaceutical Industry. Engineering.

[4] Singhal D. (2004). Drug Polymorphism and Dosage Form Design: A Practical Perspective. Adv. Drug Deliv. Rev..

[5] Steed J.W. (2010). Anion-Tuned Supramolecular Gels: A Natural Evolution from Urea Supramolecular Chemistry. Chem. Soc. Rev..

[6] Kumar D.K., Steed J.W. (2014). Supramolecular Gel Phase Crystallization: Orthogonal Self-Assembly under Non-Equilibrium Conditions. Chem. Soc. Rev..

[7] Sharma H., Kalita B.K., Pathak D., Sarma B. (2023). Low Molecular Weight Supramolecular Gels as a Crystallization Matrix. Cryst. Growth Des..

[8] Rahim M.A., Hata Y., Björnmalm M., Ju Y., Caruso F. (2018). Supramolecular Metal–Phenolic Gels for the Crystallization of Active Pharmaceutical Ingredients. Small.

[9] Smith D.K. (2024). Supramolecular Gels—A Panorama of Low-Molecular-Weight Gelators from Ancient Origins to next-Generation Technologies. Soft Matter.

[10] Contreras-Montoya R., Álvarez de Cienfuegos L., Gavira J.A., Steed J.W., Gavira A., Contreras-Montoya R. (2024). Supramolecular Gels: A Versatile Crystallization Toolbox. Chem. Soc. Rev..

[11] Zhang Q., Yan Y., Xu Y., Zhang X., Steed J.W. (2025). Selective Crystallization of Pyrazinamide Polymorphs in Supramolecular Gels: Synergistic Selectivity by Mimetic Gelator and Solvent. J. Colloid. Interface Sci..

[12] Artusio F., Castellví A., Sacristán A., Pisano R., Gavira J.A. (2020). Agarose Gel as a Medium for Growing and Tailoring Protein Crystals. Cryst. Growth Des..

[13] Mullin J.W., Mullin J.W.B.T.-C., Fourth E. (2001). Crystal Growth. Crystallization.

[14] Wang C., Zhou L., Zhang X., Yang Y., Yin Q., Roberts K.J. (2018). The Role of Solvent Composition and Polymorph Surface Chemistry in the Solution-Mediated Phase Transformation Process of Cefaclor. Ind. Eng. Chem. Res..

[15] Miller J.M., Collman B.M., Greene L.R., Grant D.J.W., Blackburn A.C. (2005). Identifying the Stable Polymorph Early in the Drug Discovery–Development Process. Pharm. Dev. Technol..

[16] Wang C., Ma C.Y., Hong R.S., Turner T.D., Rosbottom I., Sheikh A.Y., Yin Q., Roberts K.J. (2024). Influence of Solvent Selection on the Crystallizability and Polymorphic Selectivity Associated with the Formation of the “Disappeared” Form I Polymorph of Ritonavir. Mol. Pharm..

[17] Turner T.D., Corzo D.M.C., Toroz D., Curtis A., Dos Santos M.M., Hammond R.B., Lai X., Roberts K.J. (2016). The Influence of Solution Environment on the Nucleation Kinetics and Crystallisability of Para-Aminobenzoic Acid. Phys. Chem. Chem. Phys..

[18] Moris M., Van Den Eede M.-P., Koeckelberghs G., Deschaume O., Bartic C., Clays K., Van Cleuvenbergen S., Verbiest T. (2021). Solvent Role in the Self-Assembly of Poly(3-Alkylthiophene): A Harmonic Light Scattering Study. Macromolecules.

[19] Saikia B., Chen D., de Coene Y., Van Cleuvenbergen S. (2024). Organogels of FmocFF: Exploring the Solvent-Dependent Gelmorphic Behavior. Gels.

[20] Luo J., Beer T.M., Graff J.N. (2016). Treatment of Nonmetastatic Castration-Resistant Prostate Cancer. Oncology.

[21] Kassouf W., Tangury S., Aprikian A.G. (2003). Nilutamide as Second Line Hormone Therapy for Prostate Cancer After Androgen Ablation Fails. J. Urol..

[22] Surov A.O., Voronin A.P., Drozd K.V., Gruzdev M.S., Perlovich G.L., Prashanth J., Balasubramanian S. (2021). Polymorphic Forms of Antiandrogenic Drug Nilutamide: Structural and Thermodynamic Aspects. Phys. Chem. Chem. Phys..

[23] Prashanth J., Surov A.O., Drozd K.V., Perlovich G.L., Balasubramanian S. (2023). Polymorphs, Cocrystal and Hydrate of Nilutamide. CrystEngComm.

[24] Dok A.R., Legat T., de Coene Y., van der Veen M.A., Verbiest T., Van Cleuvenbergen S. (2021). Nonlinear Optical Probes of Nucleation and Crystal Growth: Recent Progress and Future Prospects. J. Mater. Chem. C.

[25] Van Cleuvenbergen S., Hennrich G., Willot P., Koeckelberghs G., Clays K., Verbiest T., Van Der Veen M.A. (2012). All Optical Determination of Microscopic and Macroscopic Structure of Chiral, Polar Microcrystals from Achiral, Nonpolar Molecules. J. Phys. Chem. C.

[26] van Cleuvenbergen S., Depotter G., Clays K., Kędziora P. (2021). Second-Order NLO Response in Chiral Ferroelectric Liquid Crystals: Molecular and Bulk Consideration. J. Mol. Liq..

[27] Galindo J.M., Tardío C., Saikia B., Van Cleuvenbergen S., Torres-Moya I. (2023). Recent Insights about the Role of Gels in Organic Photonics and Electronics. Gels.

[28] Diffraction RO Rigaku Oxford Diffraction (2014). CrysAlisPro Software System.

[29] Sheldrick G.M. (2015). Crystal Structure Solution with ShelXT. Acta Crystallogr. A.

[30] de Coene Y., Jooken S., Deschaume O., Van Steenbergen V., Vanden Berghe P., Van den Haute C., Baekelandt V., Callewaert G., Van Cleuvenbergen S., Verbiest T. (2022). Label-Free Imaging of Membrane Potentials by Intramembrane Field Modulation, Assessed by Second Harmonic Generation Microscopy. Small.

[31] Groom C.R., Bruno I.J., Lightfoot M.P., Ward S.C. (2016). The Cambridge Structural Database. Acta Crystallogr. B Struct. Sci. Cryst. Eng. Mater..

[32] Macrae C.F., Sovago I., Cottrell S.J., Galek P.T.A., McCabe P., Pidcock E., Platings M., Shields G.P., Stevens J.S., Towler M. (2020). Mercury 4.0: From Visualization to Analysis, Design and Prediction. J. Appl. Crystallogr..

