# Supplementary material for: Polymorphic Control in Pharmaceutical Gel-Mediated Crystallization: Exploiting Solvent–Gelator Synergy in FmocFF Organogels
Source: Gels. 2025 Jul 1;11(7):509. doi: 10.3390/gels11070509 (PMC12295364; doi:10.3390/gels11070509)
Supplement: Supplementary file 1 [file gels-11-00509-s001.zip › gels-3721582-supplementary.pdf]

ESI

Polymorphic Control in Pharmaceutical Gel-Mediated Crystallization: Exploiting Solvent-Gelator Synergy in FmocFF Organogels

Dong Chen[1], Koen Robeyns[2], Tom Leyssens[2], Basanta Saikia[1], Stijn Van Cleuvenbergen[1]*

1 Department of Chemistry, Molecular Imaging and Photonics, KULAK—KU Leuven, E. Sabbelaan 53, 8500 Kortrijk, Belgium; dong.chen@kuleuven.be (D.C.); bsaikia1@gmail.com (B.S.)
2 Institute of Condensed Matter and Nanosciences, Université catholique de Louvain, 1348 Louvain-La-Neuve, Belgium; tom.leyssens@uclouvain.be (L.T.); koen.robeyns@uclouvain.be (K.R.)
* Correspondence: stijn.vancleuvenbergen@kuleuven.be

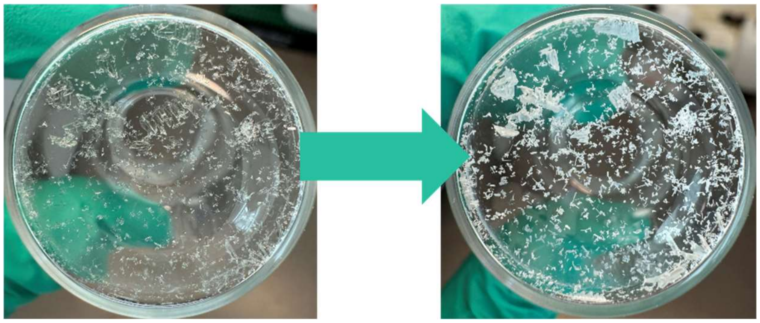

Figure S1. Optical images illustrating the crystal transformation observed for nilutamide crystallized from neat chloroform (CHL). (Left) Initially formed transparent crystals (solvate). (Right) Opaque, polycrystalline material formed after desolvation.

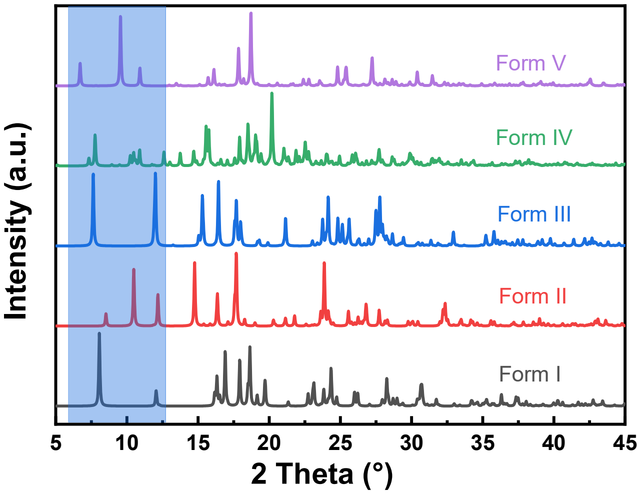

Figure S2. PXRD patterns of five reported nilutamide polymorphs (Form I to V), the blue block highlight distinguishable fingerprint peaks between 2θ value 5° and 15°. The light blue shaded region indicates the key discriminating range for nilutamide.

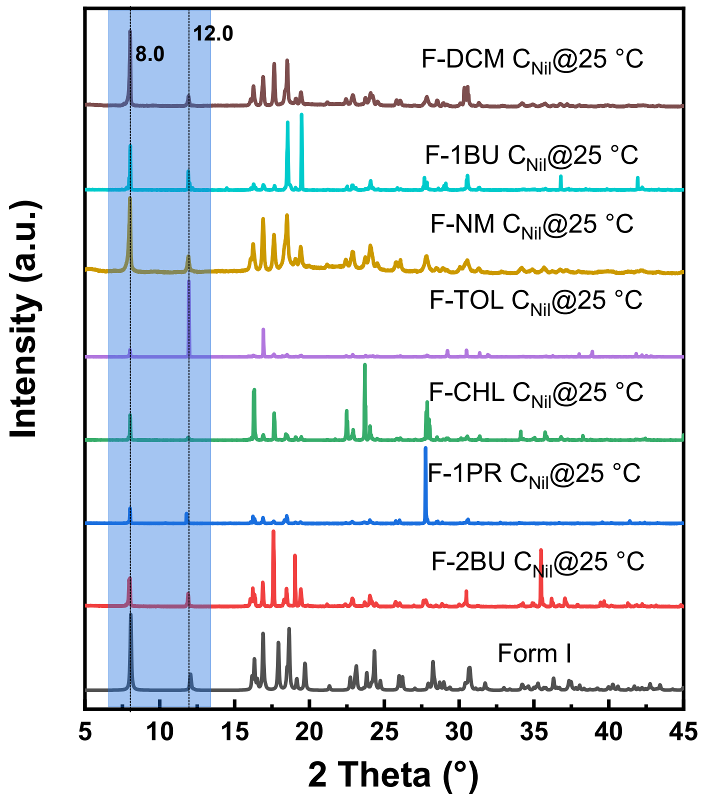

Figure S3. Powder X-ray Diffraction (PXRD) patterns of nilutamide crystals obtained from crystallization within seven FmocFF organogels under C_{Nil}@25 °C. All samples were vacuum filtered and dried in fume hood at room temperature. The light blue shaded region indicates the key discriminating range for nilutamide.

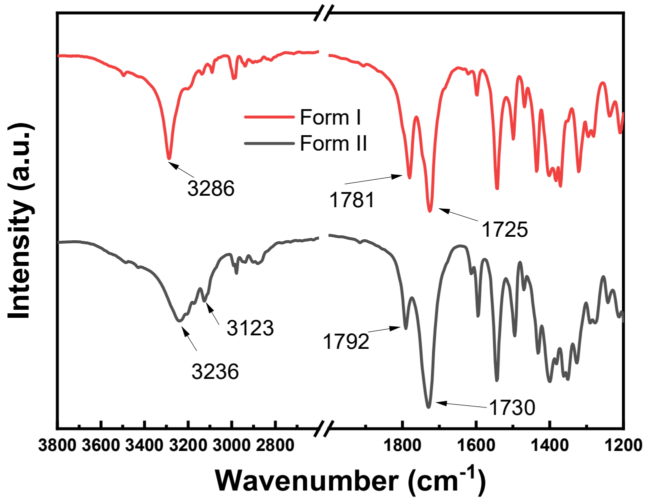

Figure S4. FTIR of nilutaimide Form I and II.

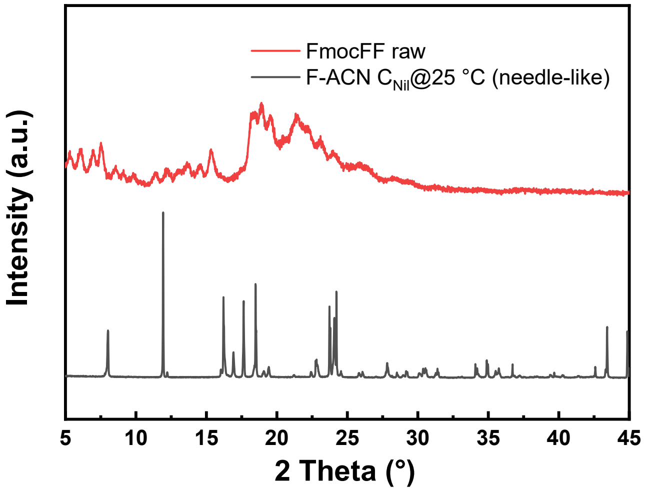

Figure S5. PXRD patterns of commercially available FmocFF and nilutamide from F-ACN C_{Nil}@25 °C.

Table S1. Empirical solubility of nilutamide in different organic solvents at 25 °C and 35 °C (mg/mL). NM = Nitromethane, 1PR = 1-Propanol, ACN = Acetonitrile, DCM = Dichloromethane, CHL = Chloroform, 1BU = 1-Butanol, 2BU = 2-Butanol, TOL = Toluene.

	**NM**	**1PR**	**ACN**	**DCM**	**CHL**	**1BU**	**2BU**	**TOL**
**25 °C**	171.67	13.34	232.50	43.07	16.58	9.60	8.99	2.32
**35 °C**	205.60	16.36	260.45	59.90	20.14	12.48	11.67	3.27
